# The Role of Prion Protein in Reelin/Dab1 Signaling: Implications for Neurodegeneration

**DOI:** 10.3390/v17070928

**Published:** 2025-06-29

**Authors:** Irene Giulia Rolle, Anna Burato, Merve Begüm Bacınoğlu, Fabio Moda, Giuseppe Legname

**Affiliations:** 1 Laboratory of Prion Biology, Department of Neuroscience, Scuola Internazionale Superiore di Studi Avanzati (SISSA), 34136 Trieste, Italy; irenegiulia.rolle@gmail.com (I.G.R.); aburato@sissa.it (A.B.); 2 Unit of Laboratory of Medicine, Laboratory of Clinical Pathology, Fondazione IRCCS Istituto Neurologico Carlo Besta, 20133 Milan, Italy; merve.bacinoglu@istituto-besta.it (M.B.B.); fabio.moda@istituto-besta.it (F.M.); 3 Department of Medical Biotechnology and Translational Medicine, University of Milan, 20133 Milan, Italy; 4 ELETTRA Sincrotrone Trieste S.C.p.A, Basovizza, 34149 Trieste, Italy

**Keywords:** prion protein, Reelin, cleavage, Dab1, neurodegeneration

## Abstract

The cellular prion protein (PrP^C^) is studied in prion diseases, where its misfolded isoform (PrP^Sc^) leads to neurodegeneration. PrP^C^ has also been implicated in several physiological functions. The protein is abundant in the nervous system, and it is critical for cell signaling in cellular communication, where it acts as a scaffold for various signaling molecules. The Reelin signaling pathway, implicated both in Alzheimer’s and prion diseases, engages Dab1, an adaptor protein influencing APP processing and amyloid beta deposition. Here, we show, using *Prnp* knockout models (*Prnp*^0/0^), that PrP^C^ modulates Reelin signaling, affecting Dab1 activation and downstream phosphorylation in both neuronal cultures and mouse brains. Notably, *Prnp*^0/0^ mice showed reduced responsiveness to Reelin, associated with altered Dab1 phosphorylation and Fyn kinase activity. Even though no direct interaction between PrP^C^ and Reelin/ApoER2 was found, *Prnp*^0/0^ neurons showed lower NCAM levels, a well-established PrP^C^ interactor. Prion infection further disrupted the Reelin signaling pathway, thus downregulating Dab1 and Reelin receptors and altering Reelin processing, like Alzheimer’s disease pathology. These findings emphasize PrP^C^ indirect role in Dab1 signaling via the NCAM and Fyn pathways, which influence synaptic function and neurodegeneration in prion diseases.

## 1. Introduction

Prion diseases, also known as transmissible spongiform encephalopathies (TSEs), are a group of fatal neurodegenerative disorders [1] characterized by sporadic, genetic, or infectious etiology [2]. Although the clinical phenotype may differ among TSEs, these disorders are characterized by shared neuropathological hallmarks, prion (PrP^Sc^) aggregation, synaptic and neuronal loss, spongiform vacuolation, and brain inflammation [3]. PrP^Sc^ is the key pathological player in TSEs, and it is defined as a “proteinaceous infectious particle”, with the unique capacity to self-replicate without requiring nucleic acids [4]. PrP^Sc^ results from the conformational change of the host-encoded cellular prion protein (PrP^C^), which is progressively converted into the pathological isoform during the disease [5]. Indeed, the α-helical-enriched structure of PrP^C^ is converted to form the β-sheet-enriched PrP^Sc^, which is partially insoluble, prone to aggregation, and partially resistant to proteases [6,7]. Although the formation and the progressive accumulation of PrP^Sc^ in the brain is thought to represent the pathogenic event leading to neurodegeneration, it is still under debate whether prion toxicity is mediated by a gain of function of PrP^Sc^, a loss of function of PrP^C^, or a combination of both mechanisms [3].

PrP^C^ is a membrane glycoprotein highly expressed in the developing and mature nervous system [8], and it is mainly exposed to the outer layer of the cell surface as a N-glycosylated glycophosphatidylinositol (GPI)-anchored protein [9,10]. Despite many efforts in the last years, the physiological functions of PrP^C^ are still elusive. However, the subcellular localization of PrP^C^ in the lipid rafts, detergent-insoluble membrane microdomains enriched in cholesterol and sphingolipids [8] and considered “hot spots” for signal transduction may indicate a possible involvement in cell signaling [11,12,13]. Indeed, PrP^C^ directly interacts with NCAM at the neuronal surface, thus regulating Fyn kinase activity and neurite outgrowth [14]. Moreover, PrP^C^ could affect PI3K and Akt/PKB activity [15,16]. Finally, PrP^C^ induces GSK3β inactivation in a caveolin/Lyn-dependent fashion [17]. All these intracellular kinases could also be regulated by yet another protein, Reelin, a large extracellular matrix glycoprotein that activates a linear downstream signaling pathway [18,19].

The binding of Reelin to two transmembrane lipoprotein receptors, apolipoprotein E receptor 2 (ApoER2) and very-low-density lipoprotein receptor (VLDLR) [20], promotes the recruitment of the intracellular adaptor protein Disabled-1 (Dab1) and its phosphorylation by Src family kinases (SFKs) [21,22,23,24]. Upon tyrosine phosphorylation, Dab1 can transduce many downstream signals. Among them, activated Dab1 could activate PI3K, leading to the activation of AKT, which, in turn, inhibits GSK3β, the main kinase involved in the phosphorylation of microtubule-associated protein Tau [21,25,26,27]. Moreover, additional signaling pathways have been recently proposed in the context of different biological settings [28].

Although the role of the Reelin signaling cascade in prion diseases has not been deeply investigated, it has been reported that APP processing and β-amyloid deposition in sporadic Creutzfeldt-Jakob disease (CJD) patients are dependent on Dab1 [29].

However, in later stages of CJD, increased levels of Reelin were observed, while low levels of Dab1 phosphorylation were found in certain subtypes of CJD. This suggests a discrepancy between Reelin expression levels and Dab1 phosphorylation status, indicating potential dysfunction in the Reelin signaling pathway in the context of prion diseases [30].

Further research is necessary to fully elucidate the intricate relationship between Reelin expression, Dab1 phosphorylation, and disease pathology in neurodegenerative conditions like prion diseases, as the expression levels of Reelin alone are not considered to be diagnostic markers [31].

Here, taking advantage of *Prnp* knockout mice (*Prnp*^0/0^) and wild-type (WT) (*Prnp*^+/+^) littermates, the expression and activation state of different components of the Reelin/Dab1 signaling pathway were evaluated in mouse brains during early postnatal days (postnatal day 4 (P4)). To better investigate the effects of the absence of PrP^C^ on Dab1 activation in response to Reelin, an ex vivo model based on primary cortical neurons obtained from *Prnp*^0/0^ and *Prnp*^+/+^ embryos was used.

Then, we used prion-inoculated WT mice to study the effects of prion infection on the Reelin pathway in vivo. We showed that prion disease strongly impairs the Reelin signaling cascade, with a prominent reduction in Dab1 protein expression.

Understanding the functional interaction between the prion protein and the Reelin/Dab1 signaling cascade is crucial, as it could reveal how alterations in this pathway may contribute to neurodegeneration and cognitive decline, potentially leading to the development of more targeted interventions for prion diseases and other neurodegenerative disorders.

## 2. Methods

### 2.1. Animals

Inbred FVB/N *Prnp*^+/+^ and PrP^C^ knockout littermate *Prnp*^0/0^ mice were used as models for PrP^C^ depletion. The day of birth was considered postnatal day (P) 0. Sex-matched P4 animals were sacrificed by decapitation; brains were extracted, immediately frozen in liquid nitrogen, and stored at −80 °C for further applications.

For neuronal primary cultures, FVB *Prnp*^+/0^ pregnant mice were used. The day of vaginal plug detection was considered embryonic day (E) 0. At E16, the pregnant females were sacrificed with CO_2_, and the embryos were extracted by caesarean cut and further processed for culture preparation.

Age- and sex-matched CD1 mice (Charles River) were used for prion infection experiments. After inoculation, mice were checked twice a week and sacrificed at specific time points or at the time of symptom onset, and the survival time was calculated. Brains were extracted, immediately frozen in liquid nitrogen, and stored at −80 °C. Animal husbandry and housing practices performed at the Besta Institute complied with the Council of Europe Convention ETS123 (European Convention for the Protection of Vertebrate Animals used for Experimental and Other Scientific Purposes; Strasbourg, 18 March 1986), Italian Legislative Decree 116/92 (Gazzetta Ufficiale della Repubblica Italiana, 18 February 1992), and 86/609/EEC (Council Directive of 24 November 1986 on the approximation of laws, regulations, and administrative provisions of the Member States regarding the protection of animals used for experimental and other scientific purposes). This study, including its ethical aspects, was approved by the Italian Ministry of Health (Permit Number: 896/2018-PR), and all efforts were made to minimize animal suffering.

### 2.2. Primary Neuronal Culture Preparation

Mouse embryonic cortical neurons were isolated and cultured as previously described [32], with some modifications. E16 embryos were collected from FVB *Prnp*^+/0^ pregnant females. Cerebral hemispheres were dissected under sterile conditions. Briefly, the cortices were separated from the midbrain, and the meninges were stripped off. Cortices from each embryo were incubated in 2 mL of 0.05% Trypsin-EDTA (Gibco, Grand Island, NY, USA) supplemented with 10 mM MgCl_2_ and 0.05 mg/mL DNaseI (Roche, Basel, Switzerland) for 10 min at 37 °C and 10 min at RT. The tissue was then homogenized by pipetting in 1 mL of neuron growth media [Neurobasal Medium (Gibco, Grand Island, NY, USA) containing 10% B27 supplement (Gibco, Grand Island, NY, USA), 1% Penicillin-Streptomycin (Gibco, Grand Island, NY, USA), 1% Glutamax (Gibco, Grand Island, NY, USA)] supplemented with 0.1 mg/mL DNaseI. Following centrifugation at 100× *g* for 5 min, pellets were resuspended in 1 mL of neuron growth media, and neurons were plated on dishes pre-coated overnight a 37 °C with poly-L-ornithine 0.05 mg/mL (Sigma-Aldrich, St Louis, MO, USA). Neurons were allowed to differentiate for 5 days in vitro (DIV) and were then treated as described below. After stimulation, cells were lysed and processed for Western Blot (WB) analysis. To detect Reelin secreted by neurons under basal conditions, neuron growth media were collected after 5 DIVs, filtered using 0.22 μm filters (Millipore, Burlington, MA, USA), and stored at −80 °C. The total protein content was measured using the bicinchoninic acid (BCA) assay (Sigma-Aldrich, St Louis, MO, USA). The Reelin content was then evaluated through WB analysis loading onto a 4–12% precast gradient gel (NuPAGE™ Novex™ 4–12% Bis-Tris Midi Protein Gels, Invitrogen, Waltham, MA, USA), using the same total protein amount for each sample.

### 2.3. Production of Reelin-Conditioned Supernatant

Reelin-containing supernatants and control supernatants were prepared as previously described [33]. Briefly, HEK-293T cells stably transfected with the expression plasmid pCrl encoding full-length mouse Reelin [20] and control cells transfected with an empty vector (mock) were grown in DMEM (Gibco, Grand Island, NY, USA) supplemented with 10% fetal bovine serum (FBS, Gibco, Grand Island, NY, USA), 1% Penicillin-Streptomycin, and 0.9 g/L G418 (Geneticin, Gibco, Grand Island, NY, USA) for 2 days to subconfluency. Cells were switched to serum-free DMEM + 1% Penicillin-Streptomycin and grown for 2 days at 37 °C in 5% CO_2_. A conditioned medium was then collected and concentrated 10-fold using 100 kDa cut-off centrifugal filters (Millipore, Burlington, MA, USA), sterile-filtered (0.22 μm filters, Millipore, Burlington, MA, USA), aliquoted, and stored at −80 °C until used. The Reelin content was analyzed by WB using anti-Reelin G10 monoclonal antibody (see Table 1).

### 2.4. Intracerebral Inoculation of Prions

Rocky Mountain Laboratory (RML) prion-infected brain homogenate was prepared at a 10% *w*/*v* concentration in phosphate-buffered saline (PBS).

For the first set of experiments, 20 μL of 10% brain homogenate was stereotactically injected into the striatum of 2-month-old outbred CD1 mice (n = 5). Non-inoculated age- and sex-matched CD1 mice were used as the control (n = 5). Inoculated mice were monitored daily for clinical signs of prion disease. All the animals were sacrificed at the terminal stage of prion infection.

For the second set of experiments, 2.5 μL of 10% brain homogenate was stereotactically injected into the hippocampus of 2-month-old outbred CD1 mice (n = 16). Non-inoculated age- and sex-matched CD1 mice were used as the control (n = 16). Pre-symptomatic animals (n = 8) were sacrificed 97 days post-inoculation (d.p.i.). Terminal-stage animals (n = 8) were monitored daily for clinical signs of prion disease and were sacrificed at the end stage of clinical prion disease.

### 2.5. Primary Neuronal Lysate Preparation

After treatment, cells were washed in PBS and lysed in CHAPS buffer (50 mM Tris-HCl, pH 7.5, 150 mM NaCl, 1 mM EDTA, 0.5% CHAPS, and 10% glycerol) supplemented with a protease inhibitor cocktail (complete mini EDTA-free protease inhibitor cocktail, Roche, Basel, Switzerland) and phosphatase inhibitors [5 mM Na_3_VO_4_, 0.5 mM NaF, and phosphatase inhibitor cocktail 2 and 3 (Sigma-Aldrich, St Louis, MO, USA)]. Samples were cleared by centrifugation at 10,000 rpm for 10 min at 4 °C, supernatants were collected, and the total protein concentration was measured using a BCA assay. Samples were then stored at −20 °C before being processed for WB analysis.

### 2.6. Tissue Homogenization for Protein Extraction

Brains from *Prnp*^0/0^ and *Prnp*^+/+^ mice were homogenized in CHAPS buffer supplemented with a protease inhibitor cocktail (complete mini EDTA-free protease inhibitor cocktail, Roche, Basel, Switzerland) and phosphatase inhibitors [5 mM Na_3_VO_4_, 0.5 mM NaF, phosphatase inhibitor cocktail 2 and 3 (Sigma-Aldrich, St Louis, MO, USA)].

Brains from prion-inoculated mice were homogenized in PBS supplemented with a protease inhibitor cocktail (complete mini EDTA-free protease inhibitor cocktail, Roche, Basel, Switzerland). After homogenization, samples were left on a rotating wheel at 4 °C for 30 min to allow complete lysis, sonicated for 30 s using a probe-type sonicator, and cleared by centrifugation (10,000 rpm, 10 min, and 4 °C). The supernatants were collected, and the total protein concentration was measured using a BCA assay. Samples were stored at −20 °C for further applications.

### 2.7. Biochemical Analysis of Brain Homogenates and Neuronal Lysates

Protein expression was analyzed using the Western Blot (WB) technique. The same total amount of protein (15–30 μg) for each sample was loaded onto a Tris-Glycine SDS-PAGE gel for protein separation after denaturation at 95 °C for 5 min in 2X loading buffer (0.1 M Tris-HCl pH 6.8, 4% SDS, 20% glycerol, 8 M Urea, 0.2 M DTT, and 0.004% bromophenol blue). Different acrylamide percentage gels were used depending on the molecular weight of the analyzed proteins. The proteins were then transferred onto a nitrocellulose membrane (GE Healthcare, Chicago, IL, USA). The membranes were blocked with 5% non-fat milk in TBST (Tris-Buffered Saline, 0.1% Tween-20) for 1 h at RT. Overnight incubation at 4 °C with primary antibodies followed. After incubation for 1 h at room temperature (RT) with secondary antibody horseradish peroxidase (HRP)-conjugated diluted in a blocking solution, the signal was detected using an enhanced chemiluminescent system (ECL, Amersham Biosciences, Chicago, IL, USA) and recorded with the digital imaging system Alliance 4.7 (UVITEC, Cambridge, UK). Densitometric analysis was performed using Uviband Analysis Software 15.0 (UVITEC, Cambridge, UK). The signals were normalized against β-Actin, which was used as the loading control. For non-phosphorylated proteins, the signals were normalized against ß-Actin or ß-III-Tubulin. For phosphorylated proteins, the membranes were developed twice to detect both the phosphorylated and the total protein (non-phosphorylated plus phosphorylated) on the same immunoblot. After the detection of the phospho-protein, the membrane was incubated with 0.01% sodium azide in 5% non-fat milk in TBST for 3 h at RT. Following several washes, the membrane was incubated overnight at 4 °C with the primary antibody against the total protein and developed as described above. The signal of the phosphorylated protein was then normalized to the signal of the total protein.

### 2.8. Immunoprecipitation of Dab1 from Brain Homogenates

Immunoprecipitation experiments were carried out using the PureProteome Protein A Magnetic Bead System (Millipore, Burlington, MA, USA). Briefly, 300 μg of total protein extracts were incubated with 2 μg of anti-Dab1 antibody (H-103, Santa Cruz Biotechnologies, Dallas, TX, USA) or with 2 μg of normal rabbit IgG (12-370, Millipore, Burlington, MA, USA), as the control, for 2 h at 4 °C on a rotating wheel. Fifty microliters of the bead suspension were used for each sample. The pre-formed antibody–antigen complex was added to the beads and then incubated for 2 h at 4 °C on a rotating wheel. The beads were then extensively washed with PBS + 0.1% Tween 20 and resuspended in 40 μL of 2X loading buffer. After 5 min of boiling at 95 °C, samples were subjected to standard immunoblotting procedures, as described above. On the same membrane used to detect immunoprecipitated protein, 30 μg of starting material (input) was loaded. The signal of the phosphorylated protein was detected using anti-phosphotyrosine 4G10 antibody (Millipore, Burlington, MA, USA), while anti-Dab1 antibody (Abcam, Cambridge, United Kingdom) was used to detect the signal of the total protein (see Table 1).

### 2.9. Co-Immunoprecipitation Experiments

Co-immunoprecipitation experiments were carried out by applying four different protocols.

In the first approach, 30 μL of Protein A/G-Sepharose (GE Healthcare, Chicago, IL, USA) was incubated with 1 mL of anti-PrP W226 hybridoma cell supernatant overnight at 4 °C on a rotating wheel. As negative controls, 14F2 anti-DISC1 antibody and a protein-free hybridoma medium (PFHM, Gibco, Grand Island, NY, USA) were used. After removing the supernatant, two washes in TBS (Tris-Buffered Saline) + 0.3% Sarcosyl were performed. The *Prnp*^+/+^ brain sample was processed in CHAPS buffer, as described above (without the sonication step), to obtain a 10% *w/v* brain homogenate, which was then diluted to 1% *w/v* in TBS + 0.3% Sarcosyl. Then, 1 mL of 1% brain homogenate was incubated with the pre-formed antibody–bead complex overnight at 4 °C on a rotating wheel. The day after, the resin was first washed in ice-cold IP1 buffer (50 mM Tris-HCl pH = 7.5, 150 mM NaCl, 1% NP-40, 0.5% DOC), then in ice-cold IP2 buffer (50 mM Tris-HCl pH = 7.5, 0.5 M NaCl, 0.1% NP-40, 0.05% DOC) and finally in ice-cold IP3 buffer (50 mM Tris-HCl pH = 7.5, 0.1% NP-40, 0.05% DOC). The beads were then resuspended in 30 μL of 2X loading buffer. After being boiled for 5 min at 95 °C, samples were subjected to standard immunoblotting procedures. On the same membrane used to detect immunoprecipitated proteins, 20 μL of starting material (input) was loaded. To perform the reverse experiment, immunoprecipitation of Reelin or ApoER2 was performed following the same protocol using 3 μg of anti-Reelin or anti-ApoER2 antibody, respectively. In these cases, normal mouse IgG (12-371, Millipore, Burlington, MA, USA) or normal rabbit IgG (12-370, Millipore, Burlington, MA, USA), respectively, were used as the negative control.

The second approach used is based on a published protocol [14], with some modifications. *Prnp*^+/+^ brain homogenates were prepared in 50 mM Tris-HCl buffer (pH 7.5), containing 0.32 M sucrose, 1 mM CaCl_2_, 1 mM MgCl_2_, and 1 mM NaHCO_3_. Samples containing 1 mg of protein were then lysed for 30 min in 50 mM Tris-HCl buffer (pH 7.5) containing 150 mM NaCl, 0.5% Triton X-100, 1% β-octyl-d-glucopyranoside, 1 mM sodium fluoride, 2 mM NaVO_4_, 0.1 mM PMSF, and an EDTA-free protease inhibitor cocktail (Roche, Basel, Switzerland). Samples were then centrifuged for 15 min at 20,000× *g* and 4 °C. Supernatants were incubated with 3 μg of anti-Reelin or anti-ApoER2 antibody, or with normal mouse IgG or normal rabbit IgG, respectively, as the negative control, overnight at 4 °C on a rotating wheel. Precipitation with 30 μL of Protein A/G-Sepharose beads for 3 h at 4 °C followed. The beads were then washed three times with RIPA buffer (150 mM NaCl, 1.0% NP-40, 0.5% DOC, 0.1% SDS, and 50 mM Tris-HCl pH = 8.0) and once with PBS. The beads were then resuspended in 30 μL of 2X loading buffer. After 5 min of boiling at 95° C, samples were subjected to standard immunoblotting procedures. On the same membrane used to detect immunoprecipitated proteins, 20 μL of starting material (input) was loaded.

In the third protocol, 1 mL of 1% *w/v Prnp*^+/+^ brain homogenate prepared in CHAPS buffer, as described above (without the sonication step), was incubated overnight at 4 °C with 3 μg of anti-Reelin or anti-ApoER2 antibody, or with normal mouse IgG or normal rabbit IgG, respectively, as the negative control. Precipitation with 30 μL of Protein A/G-Sepharose beads for 3 h at 4 °C followed. The beads were then washed three times with CHAPS buffer and resuspended in 30 μL of 2X loading buffer. After 5 min of boiling at 95 °C, samples were subjected to standard immunoblotting procedures. On the same membrane used to detect immunoprecipitated proteins, 20 μL of starting material (input) was loaded.

Finally, protein crosslinking was applied to brain samples, following the manufacturer’s instructions, before proceeding with immunoprecipitation (see Figure S1). Briefly, the *Prnp*^+/+^ brain sample was homogenized in PBS supplemented with 1 mM MgCl2 and 0.1 mM CaCl2, lysed for 40 min at 4 °C on a rotating wheel, and cleared by centrifugation (16,000 rcf, 10 min, 4 °C). Then, 1 mL of supernatant was incubated with 30 μL of 20 mM DSP (dithiobis [succinimidyl propionate], ThermoScientific, Waltham, MA, USA) for 30 min at RT on a rotating wheel (protected from light). Subsequently, 50 μL of 1M Tris pH = 7.5 was added, and the sample was then incubated for 15 min at RT on a rotating wheel. Following centrifugation (21,000 rcf, 20 min, and 4 °C), the pellet was resuspended in 800 μL of CHAPS buffer supplemented with protease and phosphatase inhibitors. After adding 50 μL of SDS 1%, the sample was subjected to a brief period of sonication. Finally, immunoprecipitation was performed following the third protocol described above.

### 2.10. Antibodies

Primary and secondary antibodies against non-phosphorylated proteins were diluted in 5% non-fat milk in TBST, while antibodies against phosphorylated proteins were used in 5% BSA in PBST. All the primary antibodies used in the present work are reported in Table 1. Secondary antibodies, goat anti-mouse and goat anti-rabbit HRP-conjugated, were from DAKO and were diluted to 1:2000 in a blocking solution. Secondary antibody (goat anti-human HRP-conjugated (ThermoScientific, Waltham, MA, USA)) was diluted to 1:5000 in a blocking solution. To avoid immunoglobulin detection, Mouse TrueBlot^®^ ULTRA (diluted to 1:30,000) and Rabbit TrueBlot^®^ (diluted to 1:10,000) secondary antibodies (Rockland Laboratories, Pottstown, PA, USA) were used to visualize Fyn and Dab1 signals, respectively, in mouse brains in standard WB (for Fyn kinase) or after immunoprecipitation (for Dab1).

### 2.11. RNA Extraction from Brain Samples and Real-Time PCR

To evaluate the expression of the Dab1 gene, total RNA was extracted from brain samples of sex-matched P4 mice. Two independent sets of samples were analyzed: four *Prnp*^0/0^ and four *Prnp*^+/+^ females, and four *Prnp*^0/0^ and four *Prnp*^+/+^ males.

For prion-infected samples, RNA was extracted from the brains of four animals for each group: WT controls of 5 months of age, WT controls of 7 months of age, RML-infected mice 3 months post-inoculation, and RML-infected mice at the terminal stage of infection.

One brain hemisphere for each sample was homogenized in TRIzol reagent (Invitrogen, Waltham, MA, USA), and total RNA was extracted following the manufacturer’s instructions. Samples were treated with an RNase-free DNase set (Qiagen, Hilden, Germany) to reduce DNA contamination and purified with an RNeasy mini kit (Qiagen). RNA quantification was performed using a NanoDrop 2000 spectrophotometer (Thermo Scientific, Waltham, MA, USA), and RNA quality was evaluated through agarose gel electrophoresis. In addition, the integrity of the 18S and 28S ribosomal bands was assessed by capillary electrophoresis using a 2100 Bioanalyzer (Agilent Technologies, Santa Clara, CA, USA).

Retrotranscription of 4 µg of RNA was performed using SuperScriptIII RT (Invitrogen, Waltham, MA, USA) and oligo-dT primer (5′-GCT GTC AAC GAT ACG CTA CGT AAC GGC ATG ACA GTG(T)24-3′). In parallel, for each sample, a non-retrotranscribed negative control (-RT) was prepared by omitting the enzyme in the reactions to evaluate genomic DNA contamination.

Expression analysis of targets of interest by quantitative reverse transcription real-time PCR (RT-qPCR) was performed using gene-specific primer pairs. All primers used for RT-qPCR are listed in Table 2. The specificity of each pair of primers for the intended target gene was verified through non-quantitative PCR, followed by agarose gel electrophoresis. RT-qPCR was carried out using 2X iQTM SYBR^®^ Green Supermix (Bio-Rad Laboratories, Inc., Hercules, CA, USA) and 400 nM of forward and reverse primers (Sigma-Aldrich, St Louis, MO, USA) on an iQ5 Multicolor Real-Time PCR Detection System (Bio-Rad Laboratories, Inc., Hercules, CA, USA). A starting amount equivalent to 1 ng of RNA was used for each reaction. After initial denaturation for 3 min at 95 °C, 40 cycles were performed at 95 °C for 15 s and 58 cycles at 60 °C for 1 min. -RT controls were included in the plates for each primer pair and sample. The expression of the target genes was normalized to the expression of two housekeeping genes: β-Actin and β-III-Tubulin. The relative expression of each gene of interest versus the housekeeping gene chosen as reference was calculated using the ΔΔCT method [36]. Significance was calculated with an unpaired Student’s *t*-test (*p*-value < 0.05).

Regarding mdab1 gene transcription, three murine Dab1 mRNA isoforms encoding proteins of 555, 217, and 271 residues have been described. Dab217 mRNA differs from the Dab555 mRNA by a consensus splice donor sequence, encodes a further 18 residues before a stop codon, and terminates with a 3′-UTR that is different from Dab555. Dab271 mRNA contains an additional exon that encodes 30 residues before a stop codon [37]. Indeed, to quantify Dab1 mRNA expression, we used two pairs of primers published in the literature: primer pair 1 (Dab1) bridges exons 2 and 3 of the Dab1 gene, which are shared by Dab555, Dab271, and Dab217, while primer pair 2 (Dab555), which bridges exons 9 and 10, was designed to detect the Dab555 mRNA only (which encodes for the 80 kDa Dab1 protein) [37].

Regarding the human Dab1 gene, its relative expression was normalized to the expression of three housekeeping genes: β-Actin, GAPDH, and RpL19. As for murine samples, the relative expression of the Dab1 gene versus the housekeeping gene chosen as reference was calculated using the ΔΔCT method [36]. Significance was calculated with an unpaired Student’s *t*-test (*p*-value < 0.05).

### 2.12. Statistical Analysis

All the results concerning Western Blot analyses of protein expression in mouse brain were compared between *Prnp*^+/+^ and *Prnp*^0/0^ samples by performing the Student’s *t*-test, setting a two-tailed distribution and two-sample unequal variance. Regarding Fyn kinase activation in brain samples, the amount of the phosphorylated protein was normalized to the total protein signal, and the ratios obtained were compared between *Prnp*^+/+^ and *Prnp*^0/0^ samples by performing the Student’s *t*-test, setting a two-tailed distribution and two-sample unequal variance. The same statistical test was applied to prion-infected samples compared to non-infected controls.

In immunoprecipitation experiments (Dab1 protein expression and phosphorylation), Dab1 expression was measured by determining the ratio of immunoprecipitated Dab1 to the starting amount of protein present in the input material. The amount of phosphorylated Dab1 was normalized to the total Dab1 signal. The results of all the experimental replicates were compared between *Prnp*^+/+^ and *Prnp*^0/0^ samples by performing a paired Student’s *t*-test, setting a two-tailed distribution.

Each experiment was performed in triplicate, unless stated otherwise.

Statistical significance: * *p* < 0.05, ** *p* < 0.01, *** *p* < 0.001, and n.s. = no statistical significance.

## 3. Results

### 3.1. Dab1 Expression Analysis

**Figure 1:**
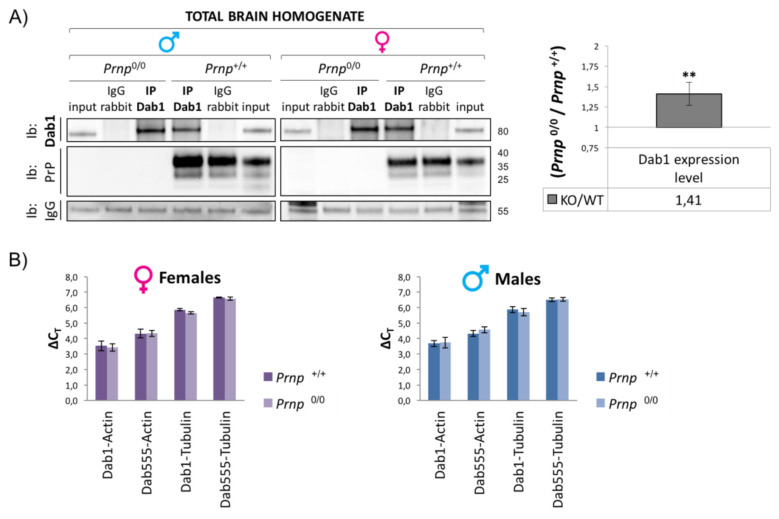
Dab1 expression and activation state in early postnatal mouse brain. (**A**) Dab1 protein levels were measured using immunoprecipitation experiments using total brain homogenates from P4 *Prnp*^0/0^ and *Prnp*^+/+^ sex-matched pups. Immunoprecipitated Dab1 was normalized to the starting amount of protein present in the input material. Sample size in each experiment: n = 1; three independent experiments for each group (males and females). Data are shown as the ratio of *Prnp*^0/0^ to *Prnp*^+/+^ ± standard error. ** *p* < 0.01. (**B**) Transcriptional analysis of Dab1 mRNA was performed using two pairs of primers: Dab1 recognizes all three murine Dab1 mRNA isoforms, and Dab555 recognizes the longer isoform only. β-Actin and βIII-Tubulin were used as reference genes. The relative expression of the Dab1 gene versus the housekeeping gene chosen as reference was calculated using the ΔΔCT method. Having observed an increase in Dab1 in *Prnp*^0/0^ mice, the expression levels of different components of the Reelin signaling pathways were subsequently analyzed. Surprisingly, Reelin, VLDLR, and ApoER2 expression levels remained unchanged in *Prnp^0/0^* brains in comparison to WT samples (Figure 2A); the phosphorylation of Fyn kinase at tyrosine 416 and, thus, its activation seemed impaired (Figure 2B).

Dab1 expression levels in *Prnp*^+/+^ and *Prnp*^0/0^ mouse brains were measured during early postnatal days to confirm the functional interaction between PrP^C^ and the Reelin pathway. *Prnp^0/0^* mice showed a 40% increased expression of Dab1 compared to WT animals in both males and females (Figure 1A). Conversely, in the same samples, Dab1 mRNA expression is not *Prnp*-dependent, as measured through transcriptional analysis of the mdab1 gene (Figure 1B).

**Figure 3:**
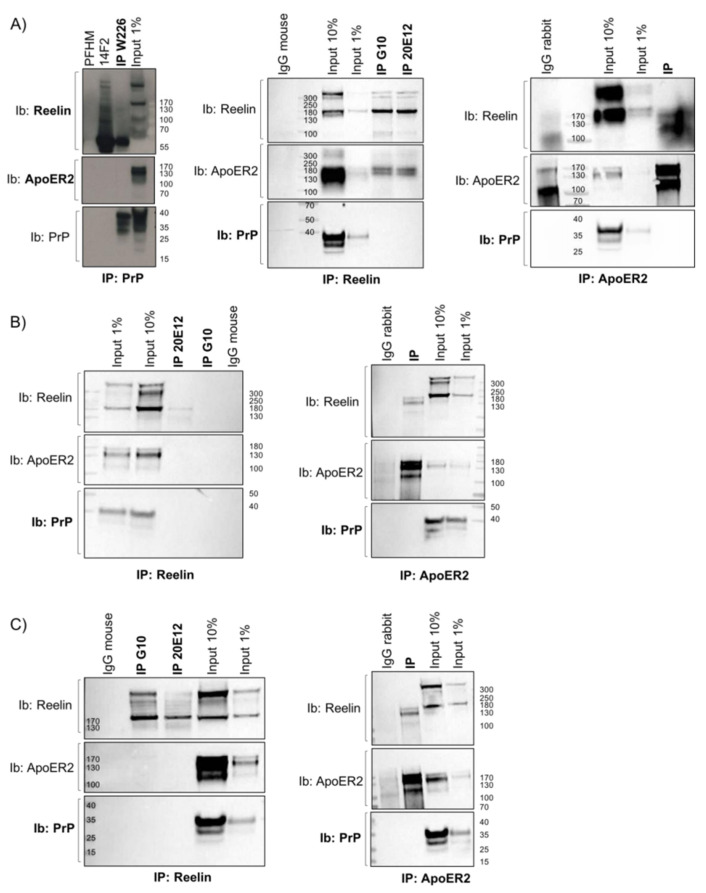
Reelin, ApoER2, and PrP^C^ are not direct interactors. PrP^C^, Reelin, or ApoER2 were immunoprecipitated from total brain homogenates from P4 *Prnp*^+/+^ mice. Immunoprecipitated samples were then immunoblotted with anti-PrP, anti-ApoER2, and anti-Reelin antibodies. Reelin signaling in ApoER2-immunoprecipitated samples is considered a positive control, and vice versa. 14F2 anti-DISC1 antibody, a protein-free hybridoma medium (PFHM), and normal mouse/rabbit IgG served as negative controls. Sample size in each experiment: n = 1; three independent experiments. The starting material (input) was also loaded on the same membrane. Three different protocols were applied, as described above (refer to the Materials and Methods section for details): (**A**) first approach; (**B**) second approach; and (**C**) third approach.

PrP^C^ on the cell membrane could play a role as a receptor or co-receptor for Reelin, thus modulating its pathway, but a direct interaction between PrP^C^ and Reelin or with ApoER2, the Reelin receptor, was ruled out, as shown through three different approaches of immunoprecipitation (Figure 3). Table 3 summarizes the interactions investigated. The lack of direct interaction between PrP^C^ and ApoER2 was further confirmed after protein crosslinking (Figure S1).

### 3.2. Stimulation with a Reelin-Conditioned Medium

Since we observed increased Dab1 protein levels in *Prnp*^0/0^ brains, Dab1 phosphorylation status in the same samples was analyzed. However, as shown in Figure S2, no significant differences in Dab1 phosphorylation between *Prnp*^0/0^ and *Prnp*^+/+^ brains were detected. Since the reliability of the results was negatively affected by the increased expression of Dab1 in *Prnp^0/0^* mice and the high variability among independent experiments, an ex vivo model was used.

Primary cortical neurons from *Prnp*^0/0^ and *Prnp*^+/+^ embryos were stimulated after 5 DIVs with a Reelin-conditioned supernatant to assess whether the absence of PrP^C^ leads to impairments in the response to Reelin. The Reelin content in the conditioned medium was evaluated by WB using an anti-Reelin-specific antibody.

**Figure 4:**
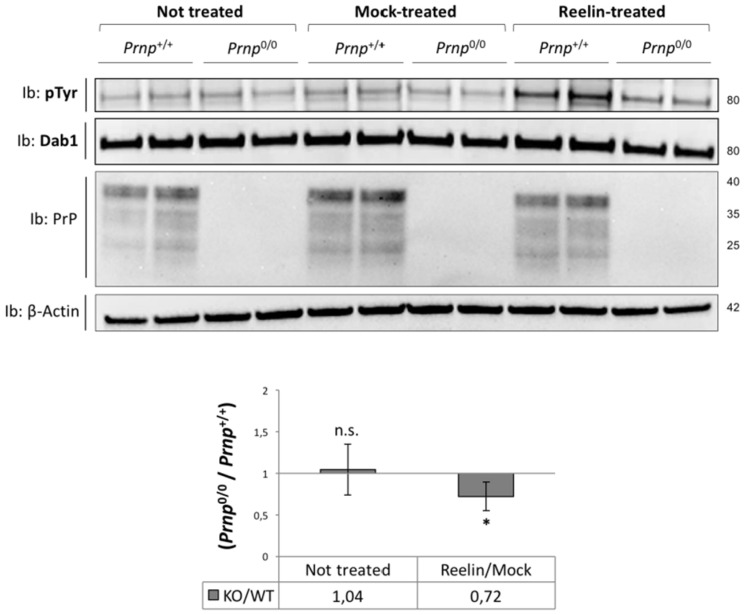
Dab1 phosphorylation (pTyr/Dab1) in primary cortical neurons upon Reelin stimulus. Dab1 phosphorylation levels were measured using WB analysis. The amount of phosphorylated Dab1 (pTyr) was normalized to the total Dab1 signal, and the ratio of Reelin- to mock-treated samples was determined. Sample size in each experiment: n = 2 (technical replicates); six independent experiments. Data are shown as the ratio of *Prnp^0/0^* to *Prnp^+/+^* ± standard error. * *p* < 0.05, n.s. = no statistical significance.

Reelin treatment was able to trigger a massive Dab1 phosphorylation in WT neurons, but Dab1 phosphorylation was not modified by the absence of PrP^C^ under basal conditions. Conversely, Dab1 activation was impaired in *Prnp*^0/0^ neurons compared to *Prnp*^+/+^ controls. Indeed, the phosphorylation of Dab1 upon Reelin stimulus was reduced by 30% in the absence of PrP^C^ (Figure 4).

To assess whether this impairment of Dab1 response is due to alterations in the Reelin signaling pathway, the expression and the activation state of different components of the cascade in *Prnp*^0/0^ and *Prnp*^+/+^ cortical neurons were checked after 5 DIVs under basal conditions. WB analysis revealed that the Reelin content in both neuron growth media (secreted protein) and neuronal lysates was unchanged in *Prnp*^0/0^ neurons compared to WT samples (Figure S3A). Moreover, ApoER2 expression levels were not modified by *Prnp* ablation. Finally, Fyn kinase expression levels were not modified in the absence of PrP^C^, while its phosphorylation showed a decreasing trend in *Prnp*^0/0^ neurons compared to WT controls (Figure S3B). As Fyn kinase activity could be modulated by NCAM, a known PrP^C^ interactor, its expression was measured in the same samples. Interestingly, NCAM expression was reduced by 15% in *Prnp^0^*^/0^ neurons in comparison to WT controls (Figure S3C).

### 3.3. Analysis of Reelin/Dab1 Signaling Pathway in Prion-Infected Mice

**Figure 5:**
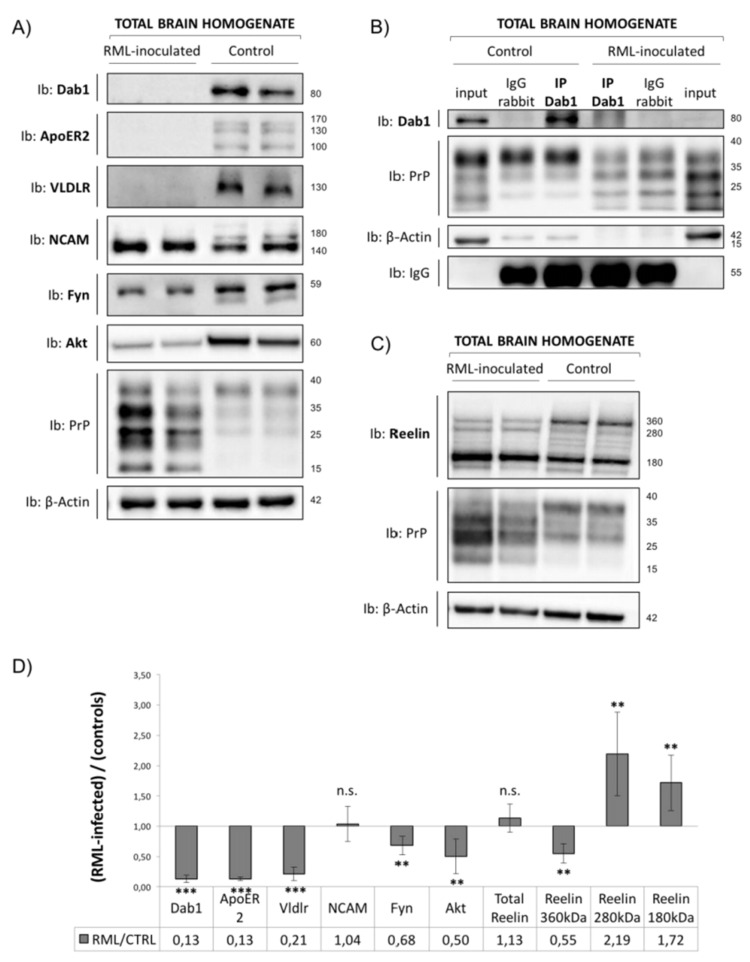
Expression of different components of the Reelin/Dab1 signaling pathway in prion-infected mice injected in the striatum. (**A**) Total brain homogenates from terminally sick mice and age-matched controls were analyzed by WB for Dab1, ApoER2, VLDLR, NCAM, Fyn, and AKT expression. β-Actin was used as the loading control. (**B**) Immunoprecipitation experiments were performed on total brain homogenates from RML-infected mice and age-matched controls to detect Dab1 protein. (C) Reelin expression was measured in total brain homogenates from terminally sick mice and age-matched controls using WB experiments. β-Actin was used as the loading control. (**D**) The graph reports the quantification analysis of the experiments shown in (**A**,**C**). Sample size: n = 5. Data are shown as the ratio of RML-infected samples to controls +- standard error to account for sample variability. ** *p* < 0.01, *** *p* < 0.001, n.s. = no statistical significance.

To investigate the effect of prion infection on the Reelin signaling cascade, we used five CD1 mice intracerebrally inoculated with the RML prion strain at the terminal stage of the disease and five non-inoculated age-matched controls. As expected, all the animals developed symptoms of prion disease about 120 days after inoculation and were sacrificed at the terminal stage of the disease. The incubation and survival times for each animal are reported (Supplementary Table S1 and Figure S4). Reelin expression levels were analyzed in brain homogenates obtained from these samples by means of immunoblotting, using anti-Reelin mouse monoclonal G10 antibody, which recognizes an epitope (amino acids 164-496) located at the N-terminal region of the protein. This antibody allows the detection of the full-length Reelin and two N-terminal fragments obtained by proteolytic cleavage, N-R6 (about 300 kDa) and N-R2 (about 180 kDa) [43]. The expression of Reelin was quantified by considering each band separately and performing normalization on β-Actin as a reference. Notably, by measuring separately each detectable band of Reelin, it was possible to appreciate pronounced changes in Reelin processing between the two groups of animals. Indeed, while full-length 360 kDa protein was reduced by almost 50% in RML-infected samples compared to controls, a concomitant 2-fold increase in both cleavage products was observed (Figure 5C). The expression levels of downstream partners of Reelin, including ApoER2 and VLDLR receptors, and the cytosolic adaptor protein Dab1 were analyzed (Figure 5A). Dab1, ApoER2, and VLDLR proteins were no longer detectable in RML-infected mice sacrificed at the terminal disease stage. This result was further confirmed through the immunoprecipitation of Dab1 protein from prion-infected samples and healthy controls (Figure 5B). Moreover, given the strong effect caused by prion infection on Dab1 protein, the expression levels of the upstream and downstream partners of Dab1 in the same samples were evaluated. Interestingly, the total expression levels of NCAM were not affected by prion infection, while in the control samples, both the 180 kDa and 140 kDa NCAM isoforms could be detected, and an enrichment in the 140 kDa isoform, with no detectable 180 kDa isoform, could be observed in prion-infected mice. As Dab1 activation is mediated by Fyn kinase [23], Fyn expression levels were measured in the same samples. Interestingly, Fyn kinase levels were reduced by more than 30% in RML-infected samples compared to non-inoculated controls. Upon tyrosine phosphorylation, Dab1 can transduce many downstream signals. Among them, activated Dab1 could activate PI3K, thus leading to the activation of AKT [21,25,26,27]. Therefore, as a downstream partner of Dab1, AKT expression levels upon prion infection were quantified, and a 50% decrease in AKT expression was observed in RML-infected samples compared to controls (Figure 5A).

All the presented findings were obtained from mice injected with the RML prion strain in the striatum. To assess the role of the injection site on the disruption of the Reelin signaling, the same analysis was performed on CD1 mice intracerebrally inoculated with the RML prion strain in the hippocampus: four of them were analyzed at a pre-symptomatic stage of the disease, and four of them were analyzed at the terminal stage of the disease. Each group of animals was compared to four non-inoculated age- and sex-matched controls and was sacrificed at the same time point. The survival times for each animal are reported (Supplementary Table S2 and Figure S5).

**Figure 6:**
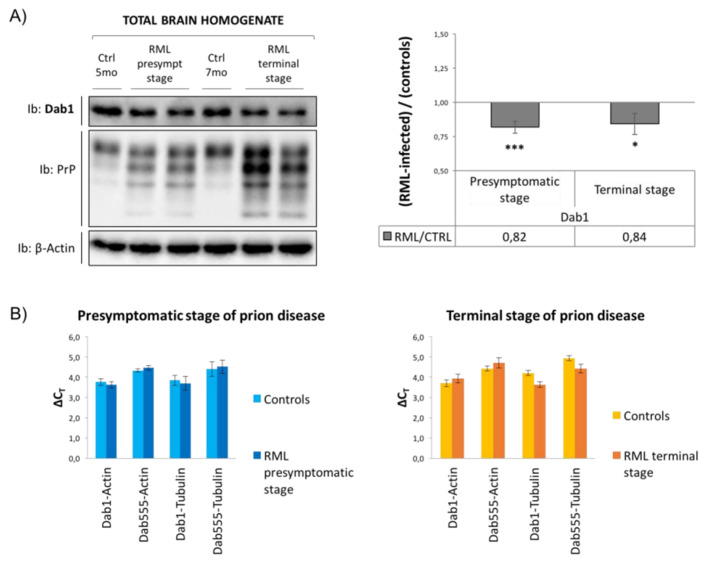
Dab1 expression in pre-symptomatic and terminally prion-infected hippocampus-injected mice. (**A**) Using WB experiments, Dab1 protein expression was measured in total brain homogenates from RML-inoculated mice, at the pre-symptomatic and terminal stages of disease, and age-matched controls. β-Actin was used as the loading control. Data are shown as the ratio of RML-infected samples to controls ± standard error. * *p* < 0.05, *** *p* < 0.001. (**B**) Transcriptional analysis of Dab1 mRNA was performed using two pairs of primers: Dab1 recognizes all three murine Dab1 mRNA isoforms, and Dab555 recognizes the longer isoform only. β-Actin and βIII-Tubulin were used as reference genes. The relative expression of the Dab1 gene versus the housekeeping gene chosen as the reference was calculated using the ΔΔCT method. Sample size: n = 4.

Given the strong effect of prion infection on Dab1 protein observed in striatum-injected mice, Dab1 expression levels were evaluated in brain homogenates obtained from hippocampus-injected samples, both at the pre-symptomatic and terminal stages of the disease. Dab1 signal was normalized to β-Actin as a reference. A decreased Dab1 protein expression in prion-infected animals was observed, both at the pre-symptomatic and terminal stages of the disease (Figure 6A). Indeed, Dab1 protein levels were reduced by 20% in RML-infected samples compared to controls, at both considered stages of the disease. However, Dab1 downregulation was less pronounced in hippocampus-inoculated animals than that observed in the striatum-infected ones, thus suggesting that the site of prion inoculation influences the degree of Reelin/Dab1 pathway disruption. Transcriptional analysis of the mdab1 gene in the same samples revealed that Dab1 mRNA expression was not modified by prion infection (Figure 6B), suggesting that the alteration in Dab1 protein expression was due to post-transcriptional mechanisms.

## 4. Discussion

PrP^C^ plays a fundamental role in modulating cell signaling, acting as a dynamic scaffold for the assembly of many different signaling molecules at the neuronal surface [8,12,13]. Indeed, it has been shown that PrP^C^ directly interacts with NCAM, promoting NCAM recruitment to lipid rafts and regulating Fyn kinase activity and neurite outgrowth [14,44]. Moreover, PrP^C^ could also affect PI3K and Akt/PKB activity [15,16], as it was shown that the recruitment of PI3K by PrP^C^ promotes cell survival, and interestingly, PI3K activity was found to be reduced in PrP-null mouse brains compared to WT animals [15]. These findings, together with the reduced phosphorylation of AKT in *Prnp*^0/0^ mice, suggest that PrP^C^ may exert a neuroprotective function, and its absence may increase susceptibility to neuronal injuries [16]. It has also been shown that PrP^C^ promotes GSK3β inactivation in a caveolin/Lyn-dependent fashion [17]. All these intracellular kinases could also be regulated by Reelin, an extracellular matrix glycoprotein that activates a linear downstream signaling pathway. As previously described, the Reelin signaling pathway is triggered by the binding of Reelin to two transmembrane lipoprotein receptors, ApoER2 and VLDLR, which promotes the clustering of receptors on the plasma membrane and the recruitment of the intracellular adaptor protein Dab1 [45,46]. Upon Fyn-mediated tyrosine phosphorylation, Dab1 is activated and can transduce various downstream signals [18].

Interestingly, it has been reported that the Reelin signaling pathway is involved in both Alzheimer’s and prion diseases. Indeed, the intracellular adaptor protein Dab1 can affect APP processing and intracellular trafficking, increasing its β-cleavage and decreasing Aβ production [47]. Also, ApoER2-Dab1 pathway disruption has been proposed as the origin of pTau-associated neurodegeneration in sporadic Alzheimer’s disease [48].

Moreover, Dab1 has been shown to influence amyloid beta deposition in sporadic Creutzfeldt–Jakob disease [49].

Considering these points, we investigated the role of PrP^C^ in regulating Reelin/Dab1 signaling, taking advantage of *Prnp*^0/0^ mice and *Prnp*^+/+^ littermate controls. Knowing that the expression of both PrP^C^ and Reelin throughout the nervous system is developmentally regulated, we carried out experiments on brain samples during early postnatal days. PrP^C^ and Reelin share a similar distribution in mammalian brain, being expressed since the early stages of brain development in cortical and hippocampal structures [45,50,51]. Reelin is highly expressed by Cajal–Retzius neurons during embryogenesis, but its expression is also maintained postnatally [18,51]. Indeed, Reelin is still highly expressed in many cells of the cortex and the hippocampus during early postnatal stages [51].

Since the intracellular adaptor Dab1 represents a key point in Reelin downstream signal transduction [18], we measured Dab1 expression levels in *Prnp*^0/0^ mouse brains. Our data revealed that Dab1 is strongly upregulated in PrP^C^-null mice, with an increase of about 40% in comparison to WT littermate controls. Conversely, transcriptional analysis of mdab1 showed that Dab1 gene expression is not influenced by the absence of *Prnp*, suggesting that the absence of PrP^C^ may affect Dab1 homeostasis at a post-transcriptional level, thus influencing its synthesis or stability. The Reelin pathway, like the majority of signaling cascades, displays a negative feedback mechanism by which the signal is switched off, and this desensitization mechanism is based on Dab1 downregulation, which, in turn, is mediated by its poly-ubiquitination and targeting to the proteasome [52]. Indeed, the disruption of the Reelin signaling cascade (by the genetic ablation of Reelin, VLDLR, ApoER2, or Fyn and Src) leads to the accumulation of Dab1 protein in vivo, while Dab1 mRNA expression remains unchanged, confirming previous results [22,23,53,54,55,56]. As *Prnp* ablation induces the same effect as Reelin signaling disruption, it may be suggested that PrP^C^ influences Dab1 protein expression by modulating the upstream signaling pathway. However, our data clearly showed that the expression of Reelin, Reelin receptors ApoER2 and VLDLR, and Fyn kinase is not altered by the absence of PrP^C^. Conversely, the activation of Fyn kinase is impaired in *Prnp*^0/0^ mice, in agreement with the previously reported evidence [14]. Fyn kinase recruitment is induced upon the binding of Reelin to its receptors, leading to Dab1 phosphorylation on tyrosine residues [23]. It is known that Dab1 poly-ubiquitination and subsequent degradation are dependent on its phosphorylation; thus, the protein tends to accumulate when its phosphorylation is impaired [37,57]. The observation that Fyn kinase activation is impaired in *Prnp*^0/0^ mice, together with Dab1 accumulation, is in line with previous findings and suggests that PrP^C^ plays a role in modulating the Reelin signaling pathway. However, co-immunoprecipitation experiments ruled out the hypothesis of a direct interaction between neither Reelin nor ApoER2.

Knowing that the activation of Fyn kinase is impaired in *Prnp*^0/0^ mice and that Dab1 protein is upregulated when its phosphorylation is impaired [37,57], we took advantage of an ex vivo model based on cultured primary cortical neurons.

In agreement with previous works [23], Reelin treatment can trigger a massive Dab1 phosphorylation in *Prnp*^+/+^ neurons. Conversely, Dab1 phosphorylation resulted in strongly impaired *Prnp*^0/0^ neurons, which appeared less responsive to Reelin stimulation than the WT mice. As already observed in brain samples, the impairment of Dab1 response is not due to alterations in the expression of Reelin or Reelin receptors, which are not modified by *Prnp* ablation in neurons under basal conditions. However, in line with our previous results on brains, Fyn kinase phosphorylation showed a decreasing trend in *Prnp*^0/0^ neurons compared to WT controls. Indeed, we hypothesized that PrP^C^ may indirectly influence the Reelin signaling pathway and, therefore, Dab1 expression and phosphorylation by modulating Fyn kinase activity, possibly through an interaction with NCAM [14,44]. In agreement with this hypothesis, we observed a 15% reduction in NCAM expression in *Prnp*^0/0^ neurons in comparison to WT controls. To understand whether this is a correlative or causative relationship, ongoing work is focused on manipulating NCAM expression to assess its impact on the Dab1/Fyn signaling axis. Data obtained from cortical neurons stimulated with Reelin suggest that PrP^C^ plays a role in modulating the Reelin signaling pathway, as the absence of PrP^C^ leads to impaired Dab1 response. Moreover, these findings suggest that PrP^C^ may influence the Reelin cascade, promoting downstream signal transduction, likely modulating Dab1 function through the NCAM/Fyn pathway. Indeed, *Prnp* ablation leads to reduced NCAM expression, impaired Fyn activation, and, ultimately, impaired Dab1 activation and downregulation. Dab1 should be downregulated in neurons to properly terminate their migration and to allow the formation of organized layered structures: a precise regulation of Dab1 levels is required to control the precise location of the migration arrest. The impairment of Dab1 downregulation is indeed predicted to cause strong cortical developmental defects [58].

Finally, as the role of the Reelin/Dab1 signaling cascade in prion diseases has not been deeply investigated, RML-inoculated mice were used as prion pathology models. Contrary to the Dab1 upregulation observed in *Prnp*^0/0^ mice, prion-infected animals display a complete abolishment of Dab1 protein expression, together with the loss of both Reelin receptors. The disruption of the Reelin signaling cascade may lead to impaired synaptic function and cytoskeleton dynamics, likely contributing to neurodegeneration. Interestingly, the site of prion inoculation seems to influence the degree of Reelin/Dab1 pathway disruption. Indeed, less pronounced Dab1 downregulation could be observed in hippocampus-inoculated animals compared to the striatum-infected ones, suggesting that specific brain regions may differ in susceptibility. In fact, comparable survival times of the two groups of animals suggest that the different effect on Dab1 expression is not due to lower levels of infectivity but may instead reflect a differential spread of prions throughout brain regions. Interestingly, reduced Dab1 expression is already pronounced in the pre-symptomatic stage of the infection, possibly suggesting that Reelin pathway disruption is an early event of prion diseases.

Similarly, for *Prnp*^0/0^ mice, the impairment in Dab1 protein expression during prion infection does not correlate with an alteration in its gene transcription, suggesting that PrP^C^ misfolding, as the absence of PrP^C^, affects Dab1 homeostasis at the post-transcriptional level.

Since the downstream signaling cascade is triggered by Reelin, we evaluated whether its expression is also impaired by prion infection. We measured the expression of Reelin in prion-infected mice, showing that the overall amount of Reelin is not modified by prion infection in comparison to control samples, while pronounced changes in Reelin processing could be observed. Indeed, while full-length 360 kDa protein is reduced by almost 50% in prion-inoculated animals compared to controls, a concomitant 2-fold increase in both cleavage products is observed, in line with previous results on AD and frontotemporal dementia patients [59,60,61]. Reelin cleavage is known to be mediated by specific proteases, including meprin-β, which cleaves Reelin at its N-terminal region, generating the N-R6 and N-R2 fragments [43]. Although our study did not directly address protease activity, the altered cleavage pattern observed in prion-infected mice may reflect the dysregulation of these enzymes. Notably, similar Reelin processing changes have been implicated in synaptic dysfunction and neurodegeneration in Alzheimer’s disease [31,61], suggesting a possible convergent pathological mechanism. This raises the hypothesis that increased meprin-β or other proteolytic activity may contribute to the observed Reelin fragmentation in prion disease.

Taken together, our findings suggest that Reelin processing may also play a role in prion diseases, thus supporting the hypothesis that the Reelin signaling pathway is involved in the pathogenesis of several neurodegenerative diseases. In particular, the enrichment in the 140 kDa isoform, with no detectable 180 kDa isoform, of NCAM observed in prion-infected mice was already shown in other disease models [62,63,64,65]. The explanation might reside in the more dynamic and signaling-related nature of the 140 kDa isoform, leading to a higher resistance to possible degradation in disease models compared to the proteolytic cleavage target NCAM-180.

Finally, since Dab1 activation is mediated by Fyn kinase [23], which, in turn, could be modulated by PrPC through NCAM [14], we evaluated the effect of prion infection on Fyn expression. Interestingly, we observed a significant reduction in Fyn expression levels in prion-inoculated animals. However, Fyn deficiency does not affect PrP^Sc^ accumulation or the clinical symptoms in mouse models of prion disease; it only moderately shortens the survival time, thus suggesting an involvement of Fyn kinase in mediating neuroprotective functions [66]. Indeed, the impairment in Fyn kinase expression observed in our prion mouse model may result in impaired neuroprotection and may contribute to neuronal loss. Increased levels of phosphorylated Fyn were previously reported both in chronically prion-infected cell lines and in animal models of prion disease [67,68]. In Alzheimer’s disease, it has been shown that increased Fyn kinase activity correlates with altered tyrosine phosphorylation of the NR2B subunit of NMDAR, which, in turn, modulates its gating properties, leading to abnormal synaptic function and neuronal loss [69].

Notably, comparing our findings on PrP-null and prion-infected mice, the Reelin/Dab1 pathway may be affected through different mechanisms. In PrP-null mice, changes are less severe and primarily related to Dab1 upregulation and impaired Fyn activation; prion-infected mice, instead, exhibit a more extensive disruption, including the loss of ApoER2, VLDLR, and altered Reelin processing. This might indicate that PrP^Sc^ induces pathological effects beyond the loss of PrP^C^ function. These specific mechanisms require further attention to be fully elucidated.

## 5. Conclusions

We investigated the role of PrP^C^ in the Reelin signaling pathway modulation, taking advantage of *Prnp*^+/+^ and *Prnp*^0/0^ mice. The expression of different components of the cascade was evaluated in the early postnatal stages of brain development, especially focusing on Dab1 protein and gene expression. Furthermore, the activation status of the signaling pathway was investigated in primary cortical neurons upon Reelin stimulus. Moreover, the role of the Reelin signaling cascade in mouse models of prion disease was investigated.

In PrP^C^ loss-of-function mouse models, we observed Dab1 protein upregulation, together with an impairment of Fyn kinase activation. Moreover, in *Prnp*-null cortical neurons, reduced NCAM expression, reduced Fyn activation, and an impairment of Dab1 activation were detected. Thus, we proposed a model in which, physiologically, PrP^C^ modulates the Reelin signaling pathway indirectly, not by physically interacting with Reelin or Reelin receptors but, more likely, through the interaction with NCAM and the regulation of Fyn kinase activity, thus influencing Dab1 phosphorylation and downstream signal transduction.

Moreover, the pathological misfolding of PrP^C^ to PrP^Sc^ was also shown to have a strong effect on the Reelin/Dab1 signaling cascade. Complete disruption of the pathway, with ApoER2, VLDLR, and Dab1 ablation, together with altered Reelin processing, was observed in RML-inoculated mice. Indeed, PrP^C^ misfolding seems to play a different role in the Reelin signaling cascade in comparison to PrP^C^ ablation. Therefore, we may speculate that the effects observed in prion-infected mouse models are not due to the loss of physiological function of PrP^C^ but, more likely, because PrP^Sc^ acquires a different pathological function.

Taken together, these findings suggest that, physiologically, PrP^C^ influences the Reelin cascade, promoting the downstream signal transduction and modulating Dab1 function through the NCAM/Fyn pathway. Pathologically, PrP^Sc^ leads to complete Dab1 signaling disruption, likely contributing to neurodegeneration. Therefore, we showed a functional interaction between PrP^C^ and the Reelin/Dab1 signaling cascade under physiological and pathological conditions.

## Figures and Tables

**Figure 2:**
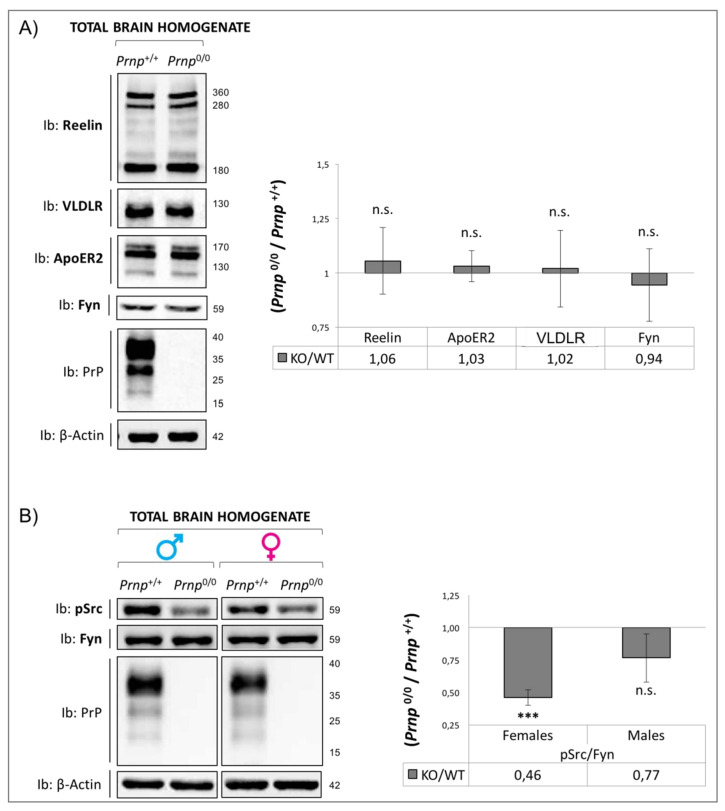
Reelin/Dab1 interplay. (**A**) Reelin, ApoER2, VLDLR, and Fyn protein levels were measured using WB experiments. Total brain homogenates from P4 *Prnp*^0/0^ and *Prnp*^+/+^ sex-matched pups were used. β-Actin was used as the loading control. (**B**) Fyn phosphorylation levels (pSrc antibody) in total brain homogenates from P4 *Prnp*^0/0^ and *Prnp*^+/+^ sex-matched pups were measured using WB experiments. The amount of phosphorylated Fyn was normalized to the total protein signal. Sample size in each experiment: n = 4; three independent experiments. Data are shown as the ratio of *Prnp*^0/0^ to *Prnp*^+/+^ ± standard error. *** *p* < 0.001, and n.s. = no statistical significance.

**Table 1:** List of the primary antibodies used for immunoblotting and immunoprecipitation.

Target	Antibody	Company/Reference	Dilution
Reelin	Mouse monoclonal G10	Millipore	1:1000
Reelin	Mouse monoclonal 20E12	Prof. Korth’s laboratory	IP
DISC1	Mouse monoclonal 14F2	Prof. Korth’s laboratory	IP
ApoER2	Rabbit monoclonal [EPR3326]	Abcam	1:1000
VLDLR	Mouse monoclonal 6A6	Millipore	1:1000
Dab1	Rabbit monoclonal [EP2248Y]	Abcam	1:1000
Dab1	Rabbit polyclonal H-103	Santa Cruz Biotechnology	1:500
Phospho-Tyrosine	Mouse monoclonal 4G10	Millipore	1:1000
Fyn	Rabbit monoclonal 04-343	Millipore	1:2000
Fyn	Mouse monoclonal (FYN-59)	Santa Cruz Biotechnology	1:1000
Phospho-Src family (Tyr416)	Rabbit polyclonal 2101S	Cell Signaling Technologies	1:1000
NCAM	Rabbit polyclonal AB5032	Chemicon International	1:1000
AKT	Rabbit polyclonal 9272	Cell Signaling Technologies	1:1000
Prion protein	Humanized D18 antibody fragment (Fab)	[34]	1:1000
Prion protein	Mouse monoclonal W226	[35]	1:1000

**Table 2:** Primer list for qRT-PCR experiments (bp = base pairs).

Target	Primer Name	Sequence (5′-3′)	Amplicon Size (bp)	Accession Number	Reference
Mouse Dab1	MoDab1_fw MoDab1_rev	GCCAAGAAAGACTCCAGGAAGA GGACCCCTTCGCCTTTAAAC	79	NM_177259.4 NM_010014.3	[37]
Mouse Dab1	MoDab555_fw MoDab555_rev	TTATGATGTGCCAAAAAGTCAACCT AGTTCTAGTTGGGTCACAGCACTTAC	51	NM_177259.4	[37]
Mouse β-Actin	MoActb_fw MoActb_rev	CACACCCGCCACCAGTTC CCCATTCCCACCATCACACC	164	NM_007393.5	[38]
Mouse βIII-Tubulin	MoTubb3_fw MoTubb3_rev	CGCCTTTGGACACCTATTC TACTCCTCACGCACCTTG	240	NM_023279.2	[39]
Human Dab1	HuDab1_fw HuDab1_rev	CACCGGGCCTTTGGATATGT GAATAACAGGTTCAGCCGCC	88	NM_021080.3	Designed
Human β-Actin	HuActb_fw HuActb_rev	AGAGCTACGAGCTGCCTGAC AGCACTGTGTTGGCGTACAG	184	NM_001101.3	[40]
Human GAPDH	HuGAPDH_fw HuGAPDH_rev	CCTGCACCACCAACTGCTTA TCTTCTGGGTGGCAGTGATG	108	NM_001289746.1	Modified from [41]
Human RpL19	HuRpL19_fw HuRpL19_rev	CTAGTGTCCTCCGCTGTGG AAGGTGTTTTTCCGGCATC	169	NM_000981.3	[42]

**Table 3:** Summary of condition and interaction results between Reelin or ApoER2 and PrP^C^. The results are shown as ✗ (no interaction) and ✓ (interaction).

IP Target	Probed For	Expectation	Result	Controls Used
PrP^C^ (W226)	Reelin	Test	✗	PFHM, anti-DISC1
PrP^C^ (W226)	ApoER2	Test	✗	PFHM, anti-DISC1
Reelin (20E12)	PrP^C^	Test	✗	Normal mouse IgG
ApoER2 (EPR3326)	PrP^C^	Test	✗	Normal rabbit IgG
Reelin (20E12)	ApoER2	Positive control	✓	Normal mouse IgG
ApoER2 (EPR3326)	Reelin	Positive control	✓	Normal rabbit IgG
DISC1 (14F2)	Reelin, ApoER2, and PrP^C^	Negative control	✗	N/A
PFHM (no Ab)	Reelin, ApoER2, and PrP^C^	Negative control	✗	N/A
Normal Mouse IgG	Reelin, ApoER2, and PrP^C^	Negative control	✗	N/A
Normal Mouse IgG	Reelin, ApoER2, and PrP^C^	Negative control	✗	N/A

## Data Availability

Available upon request.

## Supplementary Materials

The following supporting information can be downloaded at: https://www.mdpi.com/article/10.3390/v17070928/s1, Figure S1. No interaction between PrP^C^ and ApoER2 after protein crosslinking; Figure S2. Dab1 phosphorylation levels; Figure S3. Expression and activation state of different components of the Reelin/Dab1 signaling pathway in E16 primary cortical neurons under basal conditions; Table S1. Incubation time and survival time of striatum-injected animals; Figure S4. Incubation time and survival time of striatum-injected animals; Table S2. Incubation time and survival time of hippocampus-injected animals; and Figure S5. Survival time of hippocampus-injected animals.
