# Supplementary material for: The Role of Prion Protein in Reelin/Dab1 Signaling: Implications for Neurodegeneration
Source: Viruses. 2025 Jun 29;17(7):928. doi: 10.3390/v17070928 (PMC12299885; doi:10.3390/v17070928)
Supplement: Supplementary file 1 [file viruses-17-00928-s001.zip › viruses-3671772-supplementary.pdf]

Supporting Information

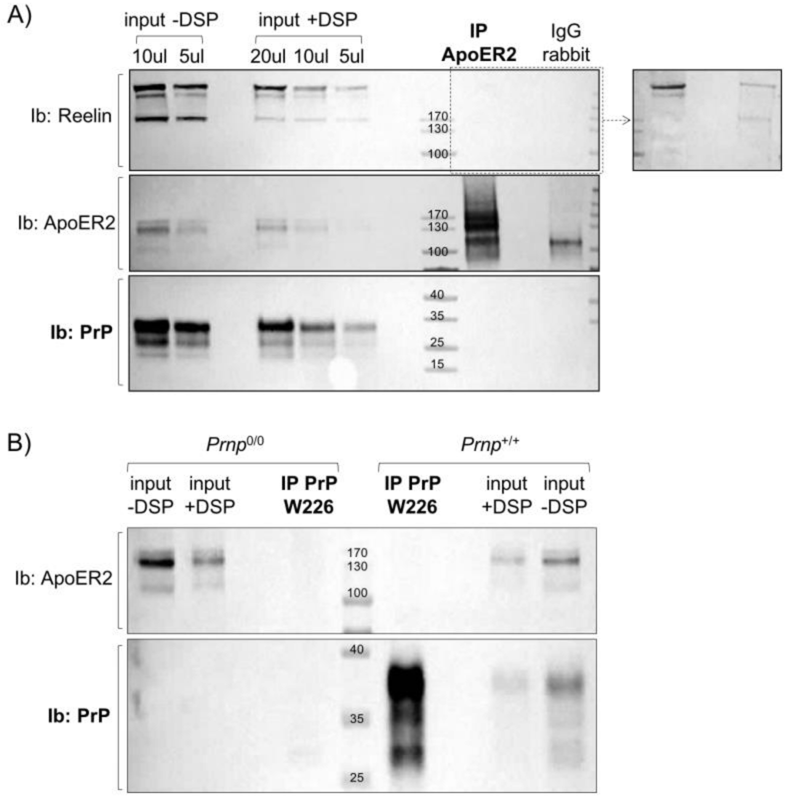

Figure S1. No interaction between PrP^C and ApoER2 after protein crosslinking. Total brain homogenates from P4 mice were treated with DSP crosslinking reagent prior to immunoprecipitation. A) ApoER2 was immunoprecipitated from $Prnp^{+/+}$ brain samples as described. Immunoprecipitated samples were then immunoblotted with anti-PrP, anti-ApoER2 and anti-Reelin antibodies. Reelin signal in ApoER2-immunoprecipitated samples served as positive control. Rabbit IgG served as negative control. Starting material (input), not treated and treated with the crosslinker, was also loaded on the same membrane. B) PrP^C was immunoprecipitated from from $Prnp^{0/0}$ and $Prnp^{+/+}$ brain samples as described. Immunoprecipitated samples were then immunoblotted with anti-PrP and anti-ApoER2 antibodies. $Prnp^{0/0}$ samples served as negative control. Starting material (input), not treated and treated with the crosslinker, was also loaded on the same membrane.

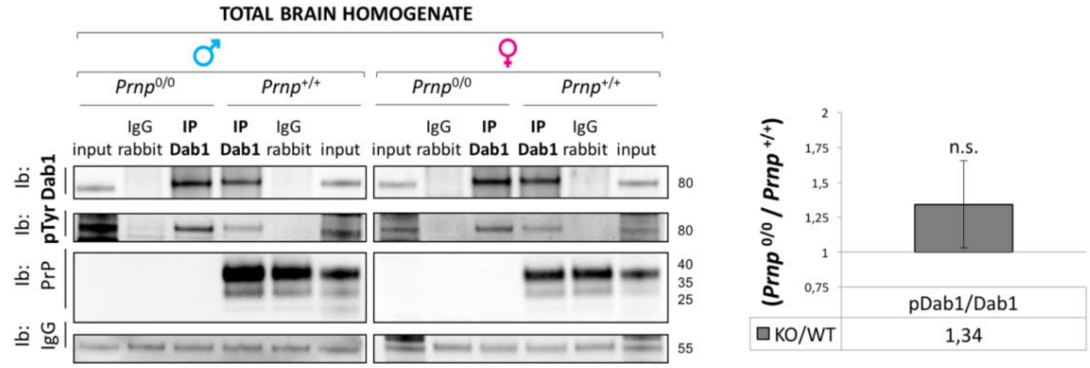

Figure S2. Dab1 phosphorylation levels. They were measured by immunoprecipitation experiments and the amount of phosphorylated Dab1 was normalized on total Dab1 signal. Data are shown as ratio between Prnp$^{0/0}$ and Prnp$^{+/+}$ ± standard error. Statistical significance: $p<0.05$, n.s. = no statistical significance.

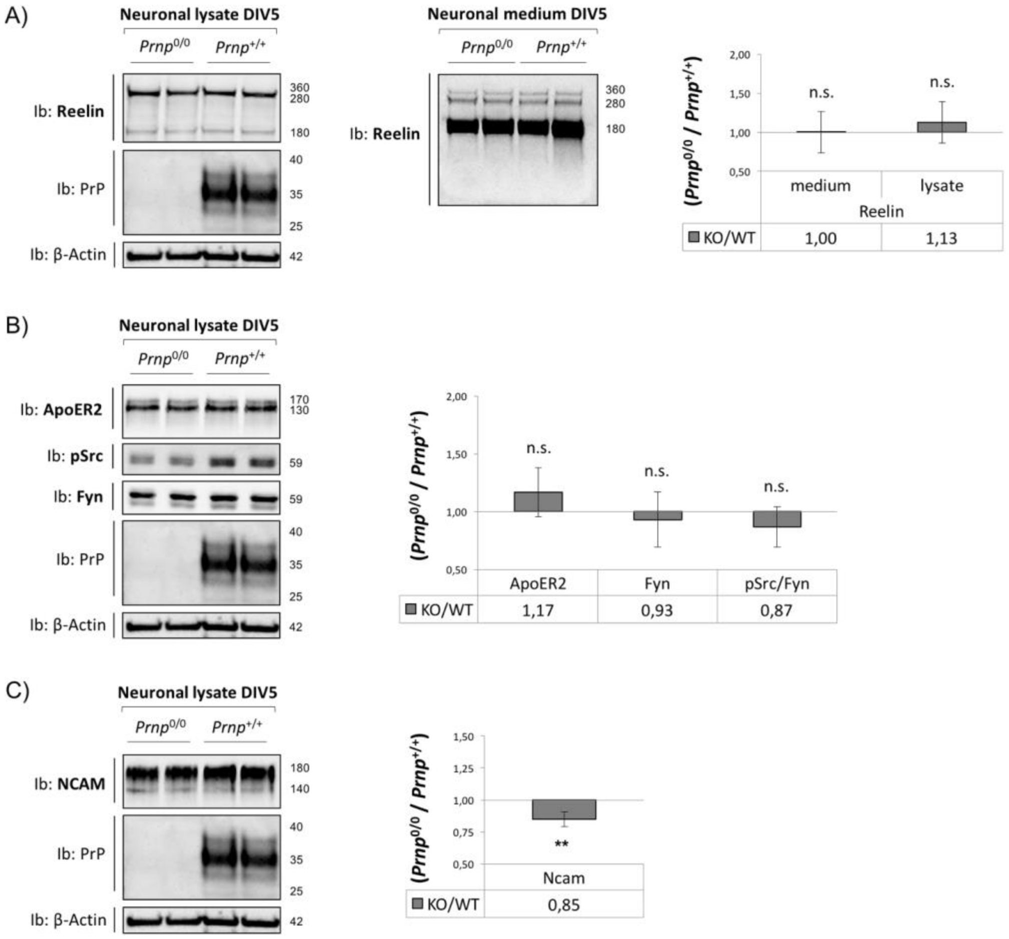

Figure S3. Expression and activation state of different component of the Reelin/Dab1 signalling pathway in E16 primary cortical neurons under basal conditions. A) Reelin expression was measured by WB experiments in primary cortical neurons from E16 $Prnp^{0/0}$ and $Prnp^{+/+}$ embryos. Protein expression was quantified in both neuronal lysates and neuronal medium after 5 DIV. β-Actin was used as loading control for lysates. B) ApoER2 protein expression and Fyn kinase expression and phosphorylation levels were evaluated by WB experiments in primary cortical neurons from E16 $Prnp^{0/0}$ and $Prnp^{+/+}$ embryos. Neuronal lysates after 5 days *in vitro* were used. β-Actin was used as loading control for protein expression. The amount of phosphorylated Fyn was normalized on the total protein signal. C) NCAM expression was measured by WB experiments in DIV5 primary cortical neurons from E16 $Prnp^{0/0}$ and $Prnp^{+/+}$ embryos. β-Actin was used as loading control. Data are shown as ratio between $Prnp^{0/0}$ and $Prnp^{+/+}$ ± standard error. *$p<0.05$, **$p<0.01$, **$p<0.001$, n.s. = no statistical significance.

LINEAGE	ANIMAL NUMBER	SEX	INOCULUM	INJECTION SITE	INCUBATION TIME (d.p.i.)	SURVIVAL TIME (d.p.i.)
Crl:CD1	C1	Male	RML	Striatum	118	132
Crl:CD1	C2	Male	RML	Striatum	118	139
Crl:CD1	C3	Male	RML	Striatum	121	140
Crl:CD1	C7	Male	RML	Striatum	125	153
Crl:CD1	C16	Female	RML	Striatum	135	160

Supplementary Table S1. Incubation time and survival time of striatum-injected animals. 8-weeks-old CD1 mice were inoculated with RML prion strain in the striatum. Incubation time and survival time for each animal is reported (d.p.i. = days post infection).

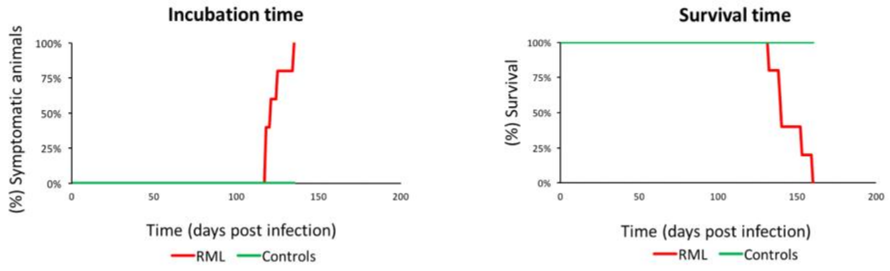

Figure S4. Incubation time and survival time of striatum-injected animals. The graphs report the incubation time (percentage of symptomatic animals in function of time, expressed as days post infection) and the survival time (percentage of alive animals in

function of time, expressed as days post infection) of 8-weeks-old CD1 mice inoculated with RML prion strain in the striatum, and not-inoculated controls.

**LINEAGE**	**ANIMAL NUMBER**	**SEX**	**INOCULUM**	**INJECTION SITE**	**DISEASE STAGE**	**SURVIVAL TIME (d.p.i.)**
Crl:CD1	1	Female	RML	Hippocampus	Presymptomatic	97
Crl:CD1	2	Female	RML	Hippocampus	Presymptomatic	97
Crl:CD1	3	Female	RML	Hippocampus	Presymptomatic	97
Crl:CD1	4	Female	RML	Hippocampus	Presymptomatic	97
Crl:CD1	5	Female	RML	Hippocampus	Presymptomatic	97
Crl:CD1	6	Female	RML	Hippocampus	Presymptomatic	97
Crl:CD1	7	Female	RML	Hippocampus	Presymptomatic	97
Crl:CD1	8	Female	RML	Hippocampus	Presymptomatic	97
Crl:CD1	9	Female	RML	Hippocampus	Terminal	134
Crl:CD1	10	Female	RML	Hippocampus	Terminal	134
Crl:CD1	11	Female	RML	Hippocampus	Terminal	134
Crl:CD1	12	Female	RML	Hippocampus	Terminal	139
Crl:CD1	13	Female	RML	Hippocampus	Terminal	145
Crl:CD1	14	Female	RML	Hippocampus	Terminal	151
Crl:CD1	15	Female	RML	Hippocampus	Terminal	147
Crl:CD1	16	Female	RML	Hippocampus	Terminal	147

Supplementary Table S2. Incubation time and survival time of hippocampus-injected animals. 8-weeks-old CD1 mice were inoculated with RML prion strain in the hippocampus. Disease stage and survival time for each animal is reported (d.p.i. = days post infection). Half of the animals were sacrificed 97 d.p.i. (pre-symptomatic stage of the disease).

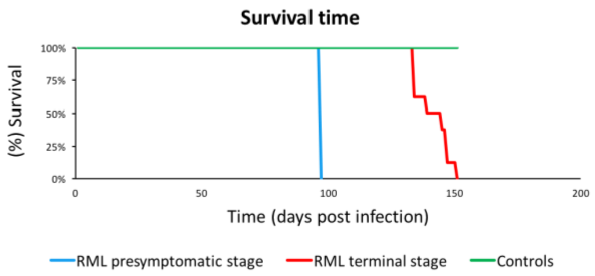

Figure S5. Survival time of hippocampus-injected animals. The graph shows the survival time (percentage of alive animals in function of time, expressed as days post infection) of 8-weeks-old CD1 mice inoculated with RML prion strain in the hippocampus, and not-inoculated controls. Half of the inoculated animals were sacrificed 97 d.p.i. (pre-symptomatic stage of the disease), and respective controls were chosen age-matched.
